# Use of benznidazole to treat chronic Chagas disease: An updated systematic review with a meta-analysis

**DOI:** 10.1371/journal.pntd.0010386

**Published:** 2022-05-16

**Authors:** Clara Crespillo-Andújar, Belén Comeche, Davidson H. Hamer, Ingrid Arevalo-Rodriguez, Noelia Alvarez-Díaz, Javier Zamora, José A. Pérez-Molina

**Affiliations:** 1 National Referral Centre for Tropical Diseases, Infectious Diseases Department, Hospital Universitario Ramón y Cajal, Madrid, Spain; 2 Instituto Ramón y Cajal de Investigación Sanitaria (IRYCIS), Madrid, Spain; 3 CIBER de Enfermedades infecciosas, Instituto de Salud Carlos III, Madrid, Spain; 4 Department of Global Health, Boston University School of Public Health, Boston, Massachusetts, United States of America; 5 Department of Medicine, Section of Infectious Diseases, Boston University School of Medicine, Boston, Massachusetts, United States of America; 6 National Emerging Infectious Diseases Laboratory, Boston University, Boston, Massachusetts, United States of America; 7 Clinical Biostatistics Unit, Hospital Universitario Ramón y Cajal, Madrid, Spain; 8 CIBER Epidemiología y Salud Pública (CIBERESP), Madrid, Spain; 9 Medical Library, Hospital Universitario Ramón y Cajal, Madrid, Spain; 10 Institute of Metabolism and Systems Research, WHO Collaborating Center for Global Women’s Health, University of Birmingham, Birmingham, United Kingdom; Babol University of Medical Science, ISLAMIC REPUBLIC OF IRAN

## Abstract

**Background:**

Approximately 6 million people worldwide are affected by Chagas disease, with many in the chronic phase of the disease (CCD). It is crucial to evaluate the effectiveness of benznidazole for CCD treatment.

**Methods/Principal findings:**

We updated a meta-analysis published in 2009 up to February 2021, including controlled trials (RCT) and prospective observational studies (OBS) that compared benznidazole vs placebo/no-treatment (P/nT). Main outcomes evaluated were clinical progression (CP) and seroreversion with subgroup analysis performed according to study design and participants’ age. Parasitological response and safety were also described.

We identified 879 articles and selected nine for inclusion (corresponding to eight studies). After adding the nine articles from the previous meta-analysis, 17 studies were analyzed corresponding to 6640 patients. The odds ratio (OR) for seroreversion in children treated with benznidazole vs P/nT was 38.3 (95%CI: 10.7–137) and 34.9 (95%CI: 1.96–624.09) in RCT and OBS, respectively. In adults the OR for seroreversion in OBS was 17.1 (95%CI: 2.3–129.1). CP was only evaluated in adults, where benznidazole did not demonstrate a beneficial effect: OR 0.93 (95%CI: 0.8–1.1) and OR 0.49 (95%CI:0.2–1.2) for RCT and OBS, respectively. Most outcomes were deemed to have a low level of certainty, except for the beneficial effect in children and the low efficacy in adults (moderate certainty).

**Conclusions:**

Benznidazole should be recommended for CCD in children, though this is only based on serological response and a moderate grade of evidence, while in adults benznidazole efficacy remains uncertain. More data on clinical efficacy of benznidazole in CCD is needed in both children and adults.

## Author summary

Chagas disease is a neglected parasitic disease, endemic in Latin America, where it poses a public health problem. Moreover, thanks to population movements, its presence has significantly increased in non-endemic areas, where it has high rates of under-diagnosis. The only two treatments currently available (benznidazole, generally the first choice, and nifurtimox) date from the 1960s and are poorly tolerated. Moreover, there is much uncertainty about their indication, dosage and benefits, especially in chronically infected adult populations. For this reason, we have update a meta-analysis published in 2009 on the effectiveness of benznidazole in chronic Chagas disease, analyzing data from 17 studies involving 6,640 patients. In the pediatric population the indication is well established thanks to some clinical trials carried out in the 1990s and the accumulated clinical experience so far. Most of the recent studies have been performed in adult populations; however, they provide low or very low certainty on the effectiveness of benznidazole except for patients with established cardiomyopathy, where benznidazole didn´t demonstrated benefit. We find that data on the treatment of indeterminate chronic infection are insufficient. Since no new drugs are expected in the near future, it would be desirable to launch trials with clinical outcomes and long follow-up periods to evaluate the efficacy of current drugs for the treatment of the indeterminate chronic Chagas disease.

## Introduction

Chagas disease, a parasitic infection caused by *Trypanosoma cruzi*, affects at least 6 million people worldwide [1]. It is endemic from the south of the United States to the south of Argentina and Chile, where people are mainly infected by several species of blood-sucking triatomine insects [2]. Chagas disease can be also transmitted through blood and blood products, transplantation, laboratory accidents, oral transmission, and vertical transmission during pregnancy or childbirth [3–6]. Thus, it can be transmitted outside of endemic areas.

Currently, 70.2 million people are at risk of infection and there are still 38,593 new infections and 12,000 deaths per year in endemic areas [1]. As a consequence of migration movements, Chagas disease has spread beyond its traditional boundaries affecting other territories, especially the United States, where 347,000 persons are estimated to be infected [7,8] and some European countries where an estimated 123,078 infected persons reside (86,618 of them in Spain) [9,10]. Chagas disease is also a concern in other countries, such as Canada (with an estimated 156,960 number of migrants from which 3.5% [5,553 persons] were estimate to be infected in 2006) and, to a lesser extent, it is also present in African, Eastern Mediterranean and Western Pacific countries [11,12].

Treatment is always indicated in the acute phase of the disease, as it has been demonstrated to improve clinical outcomes in addition to achieving parasitological clearance and seroreversion in most patients (76% to 100%) [13–15]. Without treatment, patients will develop chronic Chagas disease, and 30–40% of them will develop visceral involvement in about 20 years, with cardiomyopathy being the most common complication (14–45%) followed by megacolon and/or megaesophagus (10–20%) [16–19]. Treatment effectiveness seems to be lower in chronic Chagas disease in adult patients (2–40%) followed-up and treated in endemic areas [15,20]. In patients with established cardiomyopathy, chronic Chagas disease does not significantly reduce clinical progression of the disease [18,21].

Currently there are only two available drugs for Chagas disease treatment: benznidazole and nifurtimox. Both drugs dates from the 1960s-70s and are frequently associated with treatment discontinuation secondary to adverse effects [22–24]. Not surprisingly, it was not until August 2017 and August 2020, respectively, that the FDA has approved their use for the treatment of Chagas disease, both for pediatric use and off-label use in adults, [25,26] while in Europe is not formally approved. However, benznidazole has a better safety profile and tolerance and it is generally the first choice drug. It is administered orally in daily doses of 5 mg/kg per day in adults and 7.5 mg/kg in children, in two or three daily doses for 60 days, although in some settings only 30 days are used for adult patients [15,18,27].

The potential benefits of benznidazole in chronic Chagas disease, compared with placebo or no treatment (P/nT), was previously analysed in a systematic review and meta-analysis published in 2009 [28]. Most of the evidence in that analysis came from observational studies, especially in adult patients. Since then, there have been several well-designed, randomized, controlled trials of the treatment of chronic Chagas disease comparing benznidazole mostly with antifungal agents [21,29–31].

The availability of high quality randomized controlled trial data provides an opportunity to update the previous systematic review with evidence published during the last 11 years in order to evaluate the balance between risks and benefits of benznidazole at standard dose as compared with placebo/no-treatment in chronic Chagas disease [28]. Specifically, we aimed to assess the effectiveness of benznidazole to prevent clinical progression (cardiovascular, digestive or other clinical event or death) and to promote seroreversion after treatment.

## Materials and methods

We performed a systematic literature review, updating the previous meta-analysis performed by Pérez-Molina et al [28]. We followed the PRISMA statement to report our findings [32]. The protocol of the study is available at: DOI 10.17605/OSF.IO/TY836.

### Eligibility criteria

We included randomized controlled trials and prospective comparative observational studies using benznidazole at the standard dose compared with placebo/no-treatment for the treatment of chronic Chagas disease. We included studies with patients treated for at least 30 days or a daily fixed dose of 300 mg of benznidazole. Studies involving drugs other than benznidazole were included provided they had a benznidazole and a placebo arm. All studies using benznidazole without a placebo/no-treatment arm were excluded.

### Patient population

We included information from chronic Chagas disease patients (both adults and children) with and without visceral involvement, with a confirmed Chagas disease infection by at least two serological tests or by parasitological techniques. Studies focused on acute infections, pregnant or breast-feeding women, or immunocompromised patients (haematological malignancies, cancer, bone marrow and solid organ transplants, hypogammaglobulinemia, or HIV infection) were excluded.

### Literature search, data collection, and reporting of results

We searched Medline, EMBASE, LILACS, and Cochrane Central Register of Controlled Trials (CENTRAL) for articles published between January 2008 and February 17, 2021 with no language restrictions. A medical librarian (N.A-D) conducted the search strategies using a combination of keywords and standardized index terms (S1 Text).

We also searched on ClinicalTrials.gov to identify unpublished clinical trials. We performed a secondary search by consulting the references of the articles included and the abstracts of the most important scientific meetings on the field (CISTM, ECMID, ECTMIH, Taller de Chagas, Chagas Disease Clinical Research Platform). All scientific information (abstract and others) not published during the 3 years following their presentation at scientific congresses were excluded.

Two reviewers independently identified eligible studies (BC and CC-A) by applying the inclusion and exclusion criteria using an eligibility form. A third investigator (JAP-M) resolved disagreements. When there were some unclear aspects of the studies, we contacted the authors for clarification. BC and CC-A performed data extraction using a pre-specified data collection form. For each study arm, we collected the primary and secondary endpoints, if available: number of patients with clinical progression, seroreversion, and parasitological clearance (PCR, blood culture, and xenodiagnosis). We also collected variables related to study design and rates of treatment discontinuation.

Two reviewers (BC and CC-A) independently assessed the risk of bias for each study using the Cochrane Risk of Bias Tool for Randomized Controlled Trials [33] and the Newcastle-Ottawa scale for prospective cohort studies [34]. Reviewers judged each criterion for bias risk and resolved any disagreements in discussion with a third reviewer (JAP-M).

We summarized our findings using Summary of Findings (SoF) tables [35] and include, in a per outcome basis, ratings of certainty of evidence using the GRADE approach [36,37]. We analyzed separately the evidence from randomized controlled trials and observational comparative studies.

### Statistical analysis

We pooled the effect estimates of individual studies for the benznidazole and placebo/no-treatment groups. The meta-analysis was based on a random effect model. We used odds ratios (OR) as the measure for the pooled effect. Missing patients were considered as benznidazole treatment failure for further analysis. We performed a sensitivity analysis of the primary and secondary endpoints including only those studies with more than 60 months of follow-up taking into account the importance of the time elapsed since treatment for the cure criteria.

Heterogeneity was evaluated using the I^2^ statistic and Chi^2^ test. We regarded heterogeneity as substantial if the I^2^ statistic was > 50% or there was a low p-value (<0.10) in the Chi^2^ test for heterogeneity. If more than ten studies were available in a meta-analysis, we performed funnel plots to assess the risk of publication bias or small studies effect and complemented them with additional tests of funnel asymmetry (Egger and Begg’s tests).

We anticipated the following sources of heterogeneity to drive subgroup analyses for the primary outcomes: study design (observational versus randomized clinical trial), presence of visceral involvement versus indeterminate form, year for study initiation, participant age (children versus adults), and the country where patients were treated (endemic versus non-endemic region). The statistical significance of the between groups differences were obtained using meta-regression analyses weighted by the inverse of the standard error of the logarithm of the OR. The dependent variable in this analysis was the logarithm of the OR and independent variables, one at each time, were the a priori defined sources of heterogeneity.

All analyses were conducted using Stata version 16 statistical software (StataCorp, College Station, TX, USA).

## Results

**Fig 1:**
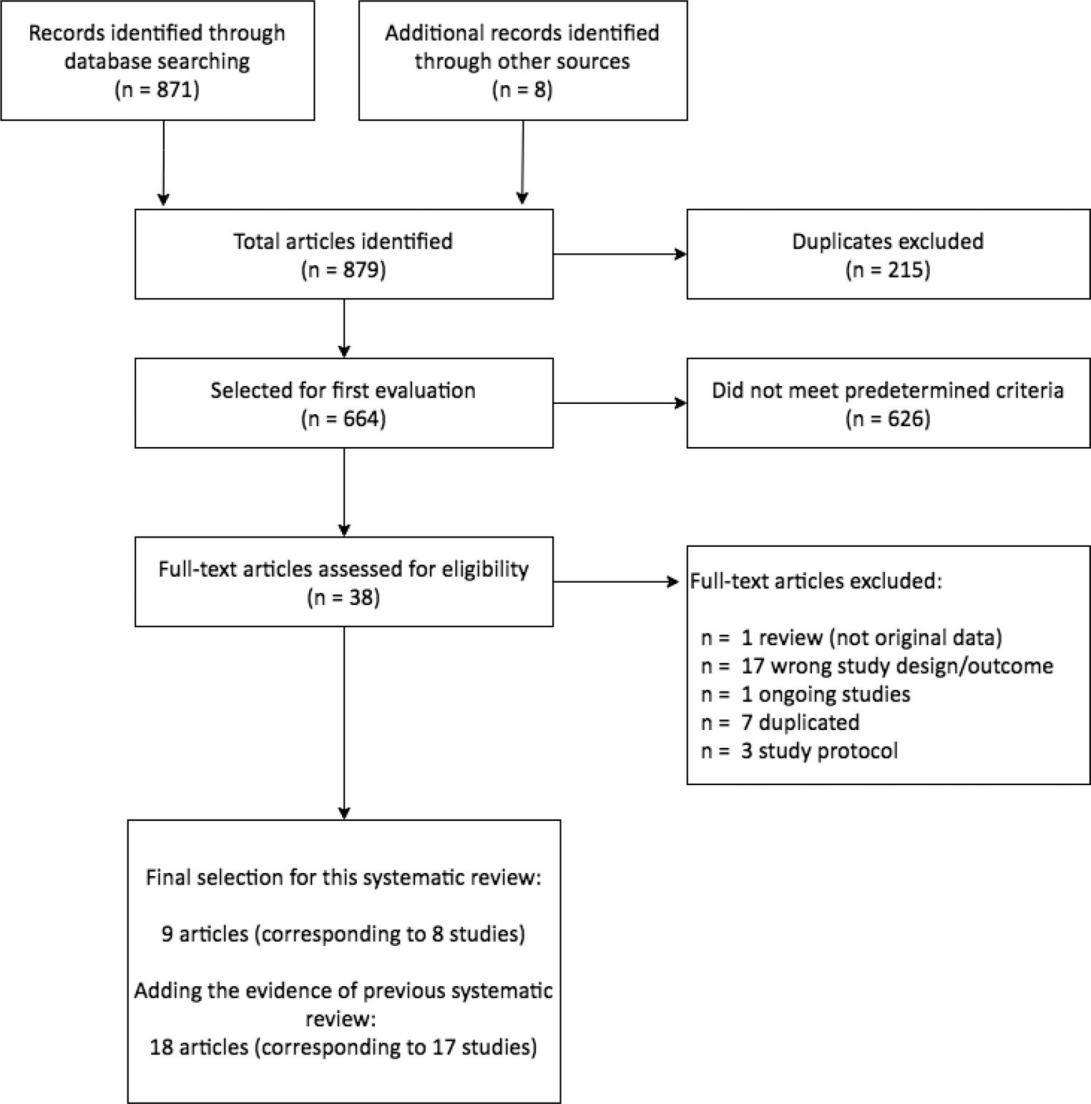
Flow diagram for selected studies.

We screened 879 articles for eligibility. After reading titles and abstracts, we excluded 626 references as they were not performed in humans, were performed on patients with acute or congenital infection, were based on other drugs than benznidazole, did not have placebo or no treatment arm, or examined diagnostic tools in Chagas disease. After a full-text reading of the remaining 38 articles, we finally selected nine articles reporting on eight studies (Fig 1).

Therefore, in this update we included data from 17 studies, including nine from the original systematic review [28] (Table 1).

**Table 1:** Summary of the characteristics of the studies included in the systematic review.

Reference Country	Study Design	Age (years) Gender	Follow-up	Clinical Form	Sample Size	Groups	Primary End Points*	Secondary End Points*	Treatment discontinuation secondary to adverse effects
1.De Andrade 1996 [38] (Galvao 2003) [39] Brazil	Randomized, double-blind, placebo-controlled CT	7–12 years F: 53%	36 m	CP	129	64: BNZ 7.5 mg/kg/day x 60 d	Negative serology values 37/64	Negative PCR values 35/58	1 case: Morbilliform exanthema
						65: PLB	Negative serology values 3/65	Negative PCR values 19/53	
2. Coura 1997 [40] Brazil	Randomized, apparently double-blind placebo-controlled CT	Adults NR	12 m	CIP	50	26 BNZ 5 mg/kg/day x 30 d		Negative XD 24/26	Toxicity led to discontinuation in 11.5% of cases.
						24 PLB		Negative XD 1/24	Toxicity led to discontinuation in 8.3% of cases.
3. Sosa Estani 1998 [41] Argentina	Randomized, double-blind placebo-controlled CT	6–12 years NR	48 m	CIP	106	55 BNZ 5 mg/kg/day x 60 d	Negative serology values 27/44	Negative XD 40/42	10% of patients had moderate adverse events that disappeared when BNZ was suspended.
						51 PLB	Negative serology values 0/44	Negative XD 21/43	Not reported
4. Catalioti 1998 [42] Venezuela	Prospective Cohort	Mean 35 y Mean 42 y F 61%	51–68 m	CP	539	74 BNZ 5 mg/kg/day x 60d	Mortality 2/74		Not reported
						465 not treated	Mortality 8/465		Not reported
5. Lauria-Pires 2000 [43] Brazil	Prospective Cohort	31–60 years NR	Mean 10 years	CP	63	17 BNZ 10 mg/kg/day x 60d		Negative PCR values 0/17	
						46 not treated		Negative PCR values 3/46	Not recorded
6. Gallerano 2000 [44] Argentina	Prospective-retrospective cohort	Mean 33.4 years F 50%	Mean 5.3 years (80 m BNZ)	CP	798	130 BNZ 4–8 mg/kg/day 45-60d	Negative serology values 3/130		10% of patients discontinued BNZ.
						668 not treated	Negative serology values 0/668		Not reported
7. Streiger 2004 [45] Argentina	Prospective Cohort	1–14 years F 60%	BNZ 4–24 years (median 3 years) Not treated 8–24 years	Early CP	88	64 BNZ 5 mg/kg/day x 30 d	Negative serology values 23/42		Two patients discontinued BNZ: one due to vomiting and the other due to cutaneous exanthema and edema
						24 not treated	Negative serology values 0/14		
8. Viotti 2006 [18] Argentina	Prospective Cohort	Mean 39 years F 54%	Mean 9.8 years	CP	566	283 BNZ 5 mg/kg/day x 30 d	Development of heart disease 12/283 Mortality 3/283 Negative serology values 32/218		Thirty-seven patients discontinued treatment due to adverse effects: allergic dermatitis (33) and GI intolerance (4).
						283 not treated	Development of heart disease 40/283 Mortality 12/283 Negative serology values 12/212		Not reported
9. De Castro 2006 [46] Brazil	Observational prospective	Mean 49 years F 57%	24 m	CP	40	27 BNZ 5 mg/kg/day x 60 d		Negative blood culture values 24/27	Three patients discontinued BNZ due to adverse reactions.
						13 not treated		Negative blood culture values 6/13	
10. Fabbro 2007 [47] Argentina	Prospective and retrospective observational	17–46 years F 68%	BNZ mean 20.6 years. Not treated mean 21.7 years	CIP	84	27 BNZ 5 mg/kg/day x 30d	Clinical progression 2/27 Negative serology values 9/27	Negative XD 27/27	6 patients from 9 with adverse effects discontinued treatment
						57 not treated	Clinical progression 9/57 Negative serology values 0/57	Negative XD 1/57	
11. Viotti 2011 [48] Argentina	Prospective Cohort	Mean 42 years F: 60%	Median 36 m	CP	142	53 BNZ 5 mg/kg/day x 30 d	Clinical progression 0/53 Negative serology values 11/53		Not recorded
						89 not treated	Clinical progression 0/89 Negative serology values 0/89		Not recorded
12. Bertocchi 2013 [49] Argentina	Prospective Cohort	Not reported for the entire population F:58%	Not reported for the entire population	Not reported for the entire population	925	545/925 BNZ 5 mg/kg/day x 30 d with a gradually increase of dose of 7 days	Negative serology values 82/545		Not recorded
						380/925 not treated	Negative serology values 25/380		Not recorded
13. Morillo 2015 [21] Argentina (559) Bolivia (357) Brazil (1358) Colombia (502) El Salvador (78)	Randomized, double-blind, placebo-controlled CT	Mean 55 years F:51%	2004–2011 (mean of 5.4 years)	Chronic phase with cardiomyopathy	2854	1431: BNZ 5 mg/kg/day 60 d	Occurrence of primary composite outcome^: 394/1431:	Negative PCR values at 2 years: 517/752	342/1429: Adverse Events Leading to Drug Interruption: Cutaneous rash (137/1429), Gastrointestinal symptoms (112/1429), Nervous system symptoms (52/1429), Serious Adverse Events Leading to Drug Interruption (119/1429)
						1423: placebo	Occurrence of primary composite outcome^: 414/1423	Negative PCR values at 2 years: 275/778	135/1422: Adverse Events Leading to Drug Interruption: Cutaneous rash (18/1422), Gastrointestinal symptoms (41/1422), Nervous system symptoms: 19/1422 20/1422: Serious Adverse Events Leading to Drug Interruption
14. Vallejo 2016 [50] Spain (93% Bolivian patients)	Randomized, open label, CT	Median 35 years F:57%	18 m	CIP	14	7: BNZ 5 mg/kg/day x 60 d		Negative PCR post treatment after 12 months: 7/7	4/7: adverse reactions that lead to treatment discontinuation in BNZ group:
						7: no treatment		Negative PCR after 12 months follow-up: 3/7	
15. Morillo 2017 [29] Argentina Chile Guatemala Colombia Mexico Spain	Randomized- single blinded placebo- controlled CT	Mean 38.6 years F:57% (BNZ) F: 23% (placebo)	180 days	CIP	120	30: BNZ 5 mg/kg/day x 60 d**		Negative PCR at day 180: 26/30	
						30: placebo		Negative PCR at day 180: 3/30	
16. Torrico 2018 [30] Bolivia	Randomized, double-blind, placebo-controlled CT	Mean 30 years F:74%	12 m	CIP	231	45: BNZ 5 mg/kg/day x 60 d		Negative PCR 37/45	Treatment discontinuation: 4/45
						47: placebo		Negative PCR: 4/47	Treatment discontinuation 0/47
17. Torrico 2021 [51] Bolivia	Randomized, double-blind, placebo-controlled CT	18–50 years Sex not reported	12 m	CIP	210	60: BZD 300mg/d 4-8w		Negative PCR: 45/52	Patients with AR that lead to TD: 3 SAE
						30: Placebo		Negative PCR: 1/30	Patients with AR that lead to TD: 0

*The definition of primary and secondary endpoints is based on that established in our systematic review and may not coincide with that assumed as a primary and secondary in each study.

** BNZ 5 mg/kg/day x 60 day until February 2009 and BNZ fixed dose of 300 mg per day and a variable duration of therapy (between 40 and 80 days) on the basis of the patient`s weight since then.

^The primary study outcome in the time-to-event analysis was the first occurrence of death, resuscitated cardiac arrest, insertion of a pacemaker or an implantable cardioverter–defibrillator, sustained ventricular tachycardia, cardiac transplantation, new heart failure, stroke or transient ischemic attack, or a systemic or pulmonary thromboembolic event.

**Abbreviations**: AR: adverse reactions; BNZ, benznidazole; CIP: Chronic Indeterminate phase; CP: Chronic phase; CT: Clinical Trial; ECG: electrocardiography; F, female; GI, gastrointestinal; m: months; NFT, nifurtimox; NR: not reported; PCR, polymerase chain reaction; PLB, placebo; pt: patient; SAE: Serious adverse event; TD: Treatment discontinuation; w: weeks; XD, xenodiagnoses; y: years

The final set of studies included eight randomized controlled trials and nine prospective observational studies including information from 6640 patients (2938 patients received benznidazole and 3702 patients taking placebo/no-treatment). Three studies were performed in children 1–14 years old, 13 studies were performed in adults, and one study did not specify the participants’ age [49].

Table 1 shows general characteristics of the studies included in the review. Most of the studies were performed in Argentina (n = 7/17; 41.2%), followed by Brazil (n = 4/17; 23.5%) and Bolivia or in Spain, with more than 90% of patients of Bolivian origin (n = 3/17; 17.6%). Information about participants’ sex was available in 13 studies with an average of 54.5% (3558/6530) female. Follow-up period and Chagas disease clinical form (cardiomyopathy, digestive involvement, neuropathy, or mixed clinical form) were available in 16 studies. Half of the studies (8/16) included only patients with the indeterminate clinical form, one (6.3%) only with cardiomyopathy and, in seven studies (43.8%), patients with and without visceral involvement. The follow-up period ranged from 3 to 552 months with a median of 36 months (p25–p75:13.5–76.2). Patients were managed with benznidazole at dose of 7.5 mg/kg/day for children and from 5–10 mg/kg/day for adult patients. A fixed dose of 300 mg per day during a variable treatment period (between 40 and 80 days) on the basis of the patient’s weight was used in two studies [21,51].

The results of the risk of bias assessment of prospective observational studies using Newcastle-Ottawa scale are shown in S2 Text. As per patient selection, most of the studies had an adequate representation of the general population exposed to Chagas disease with reasonable diagnostic and clinical evaluation prior to the inclusion of participants. Regarding comparability domain, five studies [42,45–47,49] were considered at an overall high risk of bias, while the comparability was considered adequate in only one study [18]. As per the outcome evaluation, most of the studies showed a high risk of bias because the high rates of lost to follow up. There was one study with no information of the follow-up length [49], and two with a suboptimal assessment of outcomes during the study period [42,43].

The risk of bias of randomized controlled trials was generally considered low or unclear (S1 Fig). Blinding of participants and allocation concealment was unclear in several studies. Two studies showed an unclear risk of bias in two or more dimensions [41,50]. Only one study was deemed to have an overall high risk of bias [40].

Benznidazole effectiveness, as measured by clinical progression, was reported in five studies [18,21,42,47,48] and assessed by negative serological results in eight studies [18,38,41,44,45,47–49]. As per the secondary endpoint, seven studies evaluated the response to therapy by PCR [21,29,30,38,43,50,51] and four studies used xenodiagnosis and/or blood culture [40,41,45,46]. Adverse effects to benznidazole were reported in 13 studies (76.5%), with dermatological (77%) and gastrointestinal (77%) being the most common, followed by neurological toxicities (30.8%) and laboratory abnormalities (23%). Treatment discontinuation rates associated to benznidazole toxicity was reported in half of the studies. These rates ranged from 8.8% [29] to 57.1% [50] in adults and from 1.5% to 3.12% in children (1 to 14 years old) [45].

We did not find significant heterogeneity among studied variables in the metaregression analysis. Thus, we finally performed a subgroup analysis according to the type of study and the age of the population, based on methodological and clinical decisions.

**Fig 2:**
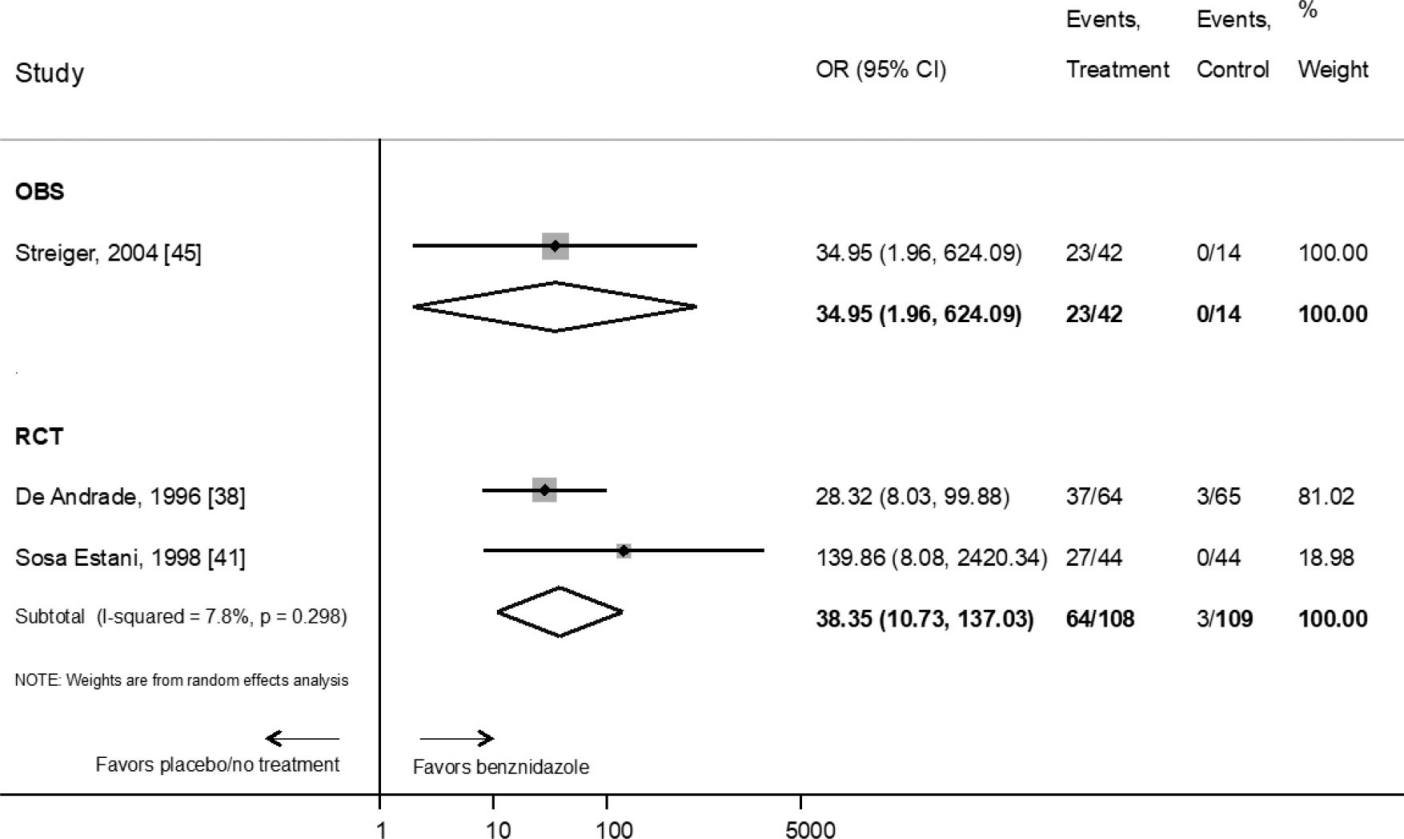
Evaluation of the effect of benznidazole compared with placebo or no treatment on the response to therapy in children according to serological primary endpoint by type of study. RCT: Randomized Clinical Trial; OBS: prospective observational study. References: [45], [38], [41].

Pooled results showed that the estimated OR for seroreversion of children receiving benznidazole compared to placebo or no treatment was 38.3 (95% CI: 10.7–137) (n = 217 patients, two clinical trials) and OR 34.95 (95%CI: 1.96–624.09) in one observational study (n = 56 patients) (Fig 2).

**Fig 3:**
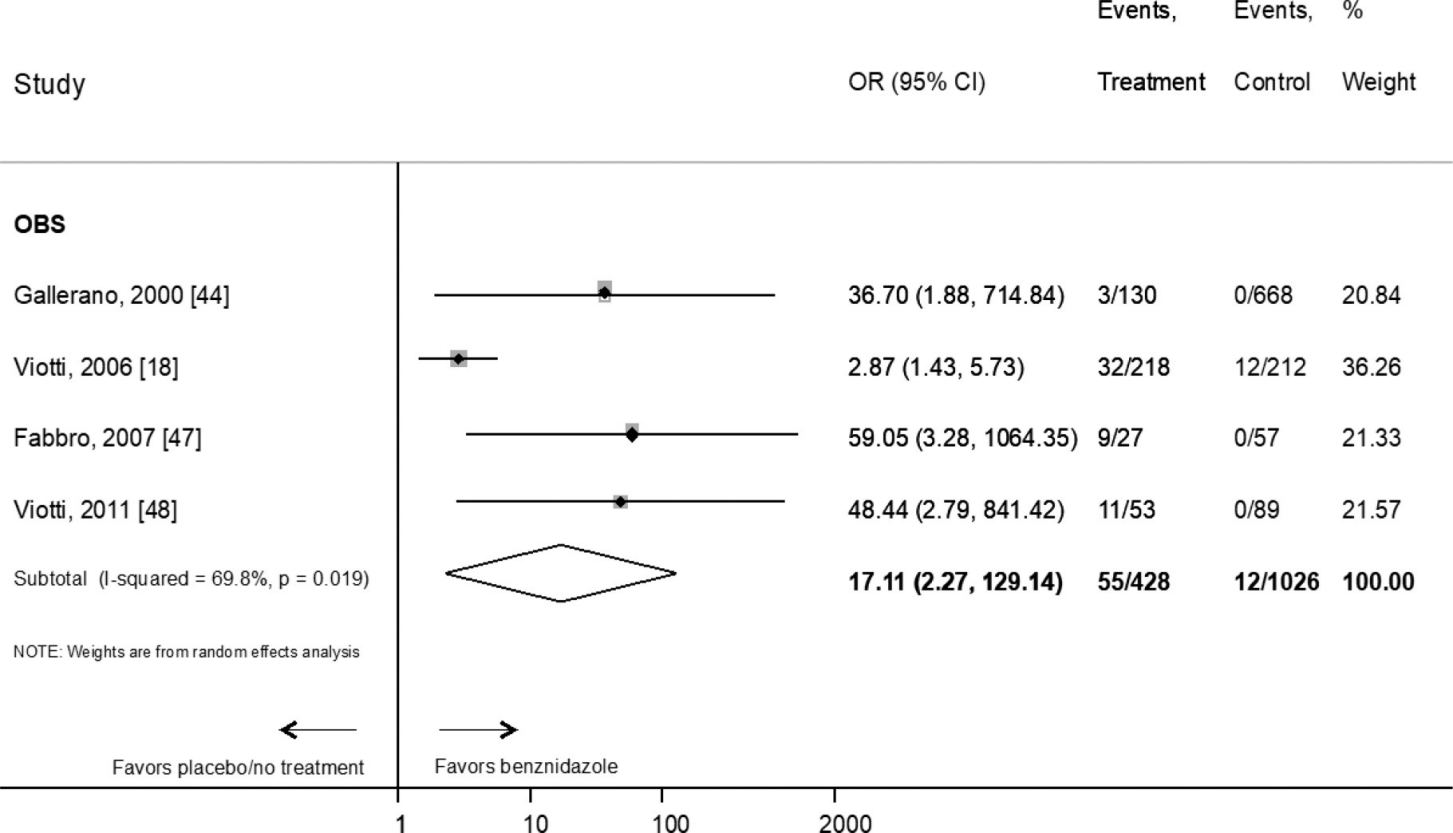
Evaluation of the effect of benznidazole compared with placebo or no treatment on the response to therapy in adults according to serological primary endpoint by type of study. RCT: Randomized Clinical Trial; OBS: prospective observational study. References: [44], [18], [47], [48].

As per the studies performed in adults, the OR for seroreversion was 17.1 (95% CI: 2.3–129.1) (n = 1454 patients, four observational studies) (Fig 3).

When only studies with more than 60 months of follow-up were considered, the OR decreased to 12.8 (95% CI: 1.3–124) (n = 1312 patients, three observational studies) (S2 Fig).

**Fig 4:**
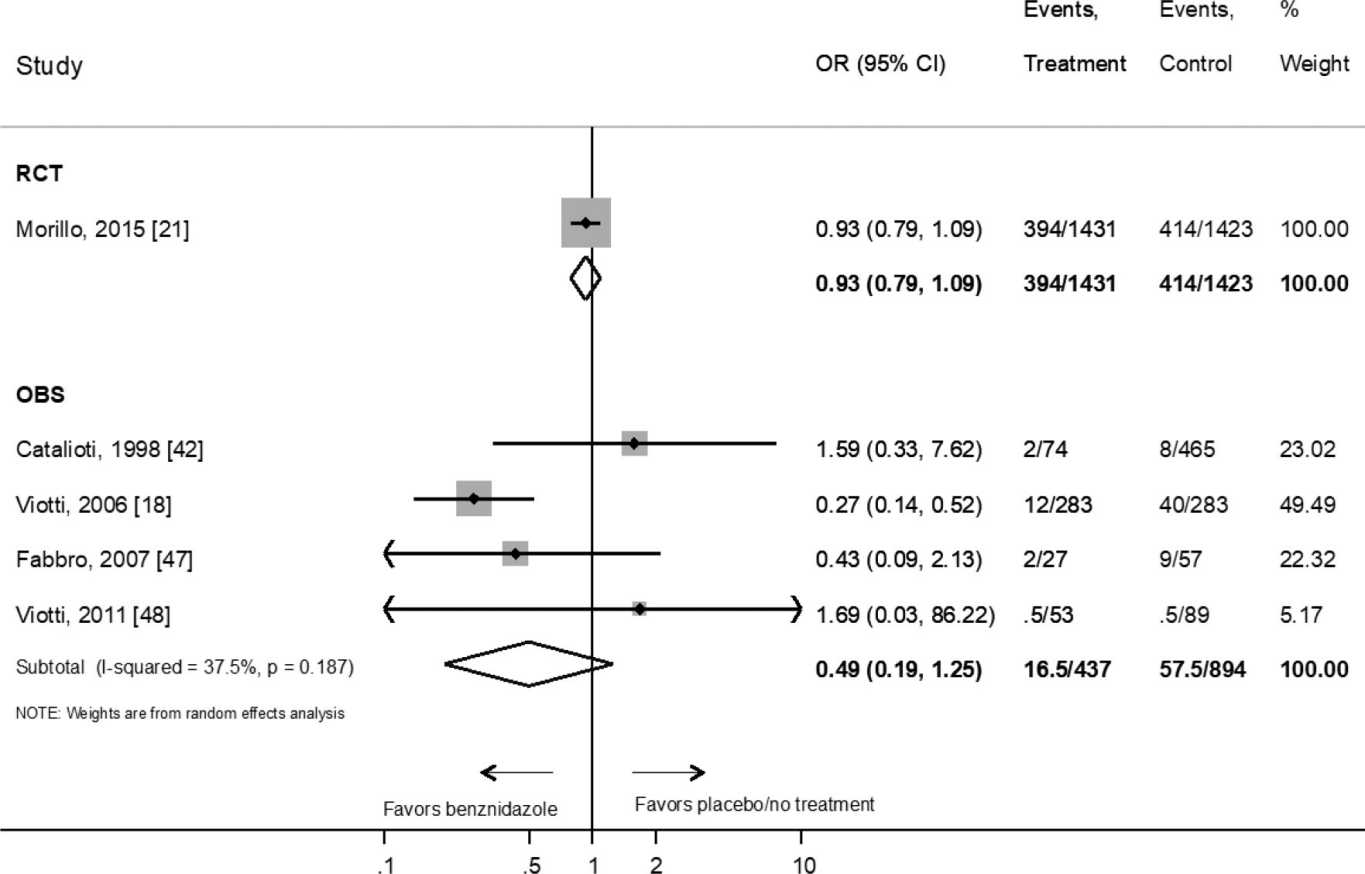
Evaluation of the effect of benznidazole compared with placebo or no treatment on the response to therapy in adults according to clinical primary endpoint by type of study. RCT: Randomized Clinical Trial; OBS: prospective observational study. References: [21], [42], [18], [47], [48].

We did not find any studies evaluating the effect of benznidazole treatment on the clinical progression of chronic Chagas disease in children. On the other hand, benznidazole efficacy in adults has only been evaluated in one randomized controlled trials which included patients diagnosed with mild to moderate cardiomyopathy [21]. The OR for progression of cardiac disease in patients treated with benznidazole versus placebo was 0.93 (95% CI: 0.8–1.1) (one RCT n = 2854 patients) (Fig 4). This lack of effect was similar when we consider data from OBS with an OR 0.49 (95% CI: 0.2–1.2) (four studies, n = 1331 patients) (Fig 4). The sensitivity analysis restricted to studies with more than 60 months of follow-up did not change overall estimations that were virtually the same (OR = 0.48; 95% CI: 0.17–1.35) (S3 Fig). Only one prospective observational studies [22], performed in a mixed group of adult patients with and without cardiomyopathy, found a significant reduction of the risk of clinical progression in those treated with benznidazole (OR: 0.27; 95% CI: 0.1–0.5) (n = 566) (Fig 4).

Regarding the secondary endpoint (parasitological clearance), there were only two randomized controlled trials performed in children, both with follow-up periods greater than 60 months, which showed a pooled OR of 6.77 (95% CI: 0.9–51.1) for parasitological clearance (S4 Fig). The OR of parasitological clearance in adults was 41.56 (95% CI: 7.25–238.15) in randomized controlled trials and 165 (95% CI: 4.5–5972) for prospective observational studies (S5 Fig). This effect decreased for randomized controlled trials (OR: 4.02; 95% CI: 3.25–4.97) and increased for prospective observational studies (OR: 899; 95% CI: 99–8152) when only studies with follow-up longer than 60 months were analyzed (S6 Fig).

We graded the certainty of evidence of the primary outcomes using GRADE approach. This grading is summarized in Table 2.

**Table 2:** Summary of outcomes and certainty of evidence (GRADE).

Outcomes	№ of participants	Certainty of the evidence (GRADE)*	Relative effect (95% CI)	Anticipated absolute effects	
				Risk with Placebo or not treatment	Risk difference with Benznidazole
SEROLOGICAL RESPONSE TO THERAPY					
RCT in children.	217 (2 CT) [38,41]	⨁⨁⨁◯ MODERATE**	**OR 38.35** (10.73 to 137.03)	28 per 1000	**493 more per 1000** (205 more to 767 more)
OBS in children	61 (1 OBS) [45]	⨁⨁◯◯ LOW	**OR 34.95** (1.96 to 624.09)	--^a^	--^a^
OBS in adults	1454 (4 OBS) [18,44,47,48]	⨁◯◯◯ VERY LOW	**OR 17.11** (2.27 to 129.14)	12 per 1000	**157 more per 1000** (14 more to 593 more)
**CLINICAL PROGRESSION**					
RCT in adults	2854 (1 CT) [21]	⨁⨁⨁◯ MODERATE	**OR 0.93** (0.79 to 1.09)	99 per 1000	**6 fewer per 1000** (19 fewer to 8 more)
OBS in adults	1331 (4 OBS) [18,42,47,48]	⨁◯◯◯ VERY LOW	**OR 0.49** (0.19 to 1.25)	64 per 1000	**32 fewer per 1000** (51 fewer to 15 more)
**PARASITOLOGICAL SECONDARY ENDPOINT**					
RCT in children	196 (2 CT) [38,41]	⨁◯◯◯ VERY LOW	**OR 6.77** (0.90 to 51.09)	417 per 1000	**412 more per 1000** (25 fewer to 557 more)
RCT in adults	1828 (6 CT) [21,29,30,40,50,51]	⨁⨁◯◯ LOW	**OR 41.56** (7.25 to 238.15)	313 per 1000	**637 more per 1000** (455 more to 678 more)
OBS in adults	187 (3 OBS) [43,46,47]	⨁◯◯◯ VERY LOW	**OR 165.12** (4.56 to 5972.95)	86 per 1000	**853 more per 1000** (215 more to 912 more)

*GRADE Working Group grades of evidence: **High certainty:** We are very confident that the true effect lies close to that of the estimate of the effect. **Moderate certainty**: We are moderately confident in the effect estimate: The true effect is likely to be close to the estimate of the effect, but there is a possibility that it is substantially different. **Low certainty:** Our confidence in the effect estimate is limited: The true effect may be substantially different from the estimate of the effect. **Very low certainty**: We have very little confidence in the effect estimate: The true effect is likely to be substantially different from the estimate of effect

**In the Sosa-Estani 1998 study, there is no information regarding the generation of the randomisation sequence, allocation concealment or blinding of outcome assesment, so we consider that there may be a risk of selection and performance bias. Regarding Andrade 1996, there are no considerable biases to downgrade the quality if the evidence.

**Abbreviations:** RCT: Randomized controlled trials, OBS: Observational studies

a. RR can not be calculated because the number of events with placebo or not treatment is cero.

**Patient or population**: Patients with chronic Chagas disease

**Intervention**: Benznidazole. **Comparison**: Placebo or not treatment

Certainty of evidence has been very variable due to several factors such as methodological issues, studies’ length of follow-up, representativeness of the studied population, rate of losses to follow-up and imprecision in the meta-analysis.

## Discussion

Even though more than 110 years have passed since the discovery of Chagas disease and more than 50 years since the availability of active drugs against *T. cruzi*, some controversy remains. Even in the absence of a formal randomized controlled trials, there is a general agreement on the efficacy of benznidazole in acute infection and reactivation disease based on evidence from studies performed in the late 1960s and 1970s, and the accumulated experience so far [15,52].

Regarding chronic Chagas disease treatment with benznidazole in children, the certainty of evidence is moderate, with the indication established based mainly on the results of randomized controlled trials conducted in the 1990s, which used seroreversion as a surrogate marker of therapeutic response. The high rates of seroreversion and the clinical practice during decades had supported this indication, as reflected the WHO Guidelines for the diagnostic and treatment of Chagas disease [15,52,53]. Furthermore, children are currently the only patients for which the US FDA has approved benznidazole for the treatment of Chagas disease in the USA [25]. Nevertheless, the certainty of the evidence is moderate to low, whether randomized controlled trials [38,41] or prospective observational studies studies [45] are considered (Table 2). Unfortunately, our estimations for seroreversion in children treated with benznidazole vs placebo/no-treatment are imprecise (wide confidence interval ranges) and have not changed from our previous meta-analysis as there has been no new evidence to include. Thus, to increase the degree of certainty concerning this indication, it would be desirable to conduct studies (as for example, prospective cohorts compared with historical controls and healthy children) with clinical outcomes in children treated with benznidazole.

In adult populations with chronic Chagas disease, there is a significant debate regarding indications for benznidazole as its benefits depends on individual factors (as visceral involvement), adding this to a poor tolerability in this population [21,22]. The time needed to achieve seroreversion is challenging and thus, conducting studies with such extended follow-up is the main limitation. Adults may have a lower frequency of seroreversion than children because of the long time-lapse from infection to treatment, leading to lower treatment efficacy. The WHO Guidelines provides a conditional recommendation in adults with chronic Chagas disease without specific organ damage, based on low certainty regarding the effects of the intervention [15]. Nevertheless, after benznidazole approval by the US FDA, the monthly average of treated patients increase from five persons to 13 (comparing all the previous period to the 9 months following its commercialization), with 90% of these patients being adults, despite that the FDA’s indication was only for paediatric use [54]. In addition, it is expected that chronic Chagas disease would progressively increase its relevance in endemic and non-endemic areas as a consequence of the empowerment of affected populations [55], better knowledge of the disease by health workers [56], and better access to diagnosis and treatment [57]. Therefore, it is mandatory to establish a common position on the indication for treatment in this population.

The efficacy of benznidazole in avoiding clinical progression has only been formally evaluated in adults. In the case of children with chronic Chagas disease, the WHO Guidelines establish a low certainty of evidence for the treatment regarding clinical outcomes and a strong recommendation based on the experts’ consensus that serological negativization is equivalent to a therapeutic response [15]. Benznidazole demonstrated no effect in reducing the progression of heart disease in a large, randomized placebo-controlled clinical trial performed in adult population with mild to severe cardiomyopathy (Fig 4) [21]. Certainty of evidence was considered moderate for benznidazole’s effect on this outcome because this study did not include the entire clinical spectrum of Chagas disease in adults (Table 2) [21]. However, this study provided important evidence that benznidazole was not valuable for patients with heart disease. Similarly, WHO Guidelines do not recommend the treatment for adult patients with chronic Chagas disease with specific organ damage based on moderate certainty regarding the effects of the intervention [15]. Regarding observational studies, benznidazole demonstrated only a marginal effect (Fig 4) with a very low degree of certainty. The significant risk of bias limited the degree of certainty resulting from the different proportion of visceral involvement among study participants, non-homogeneous length of follow-up, and inconsistency of results between studies (Table 2). Thus, randomized controlled trials in adults without visceral involvement are needed to determine whether benznidazole is helpful in this indication.

This meta-analysis’s main limitations are a consequence of the particular characteristics of the studies included and the different behaviour of the disease in children and adults. The fact that Chagas disease is a neglected disease has limited the generation of high-quality evidence over time. The long latency period of chronic Chagas disease until the development of visceral involvement, competing risks in long periods of follow-up, and lack of surrogate markers are also barriers that make research challenging. In addition, parasitological outcomes cannot be considered a reliable surrogate marker for benznidazole’s therapeutic efficacy. Blood cultures and xenodiagnosis have very low sensitivity [58] and have fallen into disuse. On the other hand, PCR techniques are much more sensitive, but, for patients with visceral involvement they have not been demonstrated to be predictive of clinical response [21].

In recent years the trypanocidal effect of other drugs, such as E1224 (ravuconazole prodrug) [30], posaconazole [29,31], and fexinidazole (ClinicalTrials.gov Identifier: NCT03587766), has been tested in clinical trials. The impact on the tolerability of various benznidazole administration schemes has also been analyzed [59–61]. Unfortunately, these randomized controlled trials have short follow-up periods and use PCR as the primary outcome, whose role after treatment is mainly as a marker of therapeutic failure, until more information becomes available. The authors of a meta-analysis comparing the efficacy and safety of a fixed vs an adjusted dose of benznidazole, did not find direct evidence comparing both schemes. Nevertheless, through indirect comparisons, they find a low to very low certainty of evidence for clinical outcomes and moderate for the efficacy (positive PCR) and safety outcomes (drug discontinuation, peripheral neuropathy and mild rash) [61]. As in our meta-analysis, they find serological response that favors the use of benznidazole vs placebo (RR 0.88; IC95% 0.84–0.93) [61]. So far, no new drug has demonstrated sufficient efficacy, nor the alternative benznidazole schemes improvement of tolerability, to be included in current therapeutics.

Our meta-analysis shows a benefit of treating chronic Chagas disease with benznidazole (with a moderate degree of certainty) in achieving seroreversion in children. It would be helpful to have studies based on clinical outcomes to better understand the drug’s efficacy profile given the lack of information on this context. As for adults, benznidazole has not demonstrated efficacy for patients with cardiomyopathy (moderate certainty). On the other hand, the quality of evidence for patients without visceral involvement is poor, with benznidazole demonstrating a marginally positive effect in the best case. Longer follow-up RCTs with adequate designs in adults without visceral involvement are needed to know whether benznidazole is helpful in this indication and to identify which patients would benefit from treatment. Considering that there will be no new drugs in the short term, early diagnosis, treatment in the early chronic phase and of women of childbearing potential are essential to maximizing the current drugs’ benefits.

## Supporting information

S1 Text Search strategy. (DOCX)

S2 Text Newcastle-Ottawa quality assessment scale for cohort studies. (DOCX)

S1 Fig Summary of risk of bias in clinical trials. References: [40], [38], [21], [29], [41], [30], [51], [50].
(TIF)

S2 Fig Evaluation of the effect of benznidazole compared with placebo or no treatment on the response to therapy in adults according to serological primary endpoint by type of study (only in those with >60 months follow-up period). OBS: prospective observational study. References: [44], [18], [47].
(TIF)

S3 Fig Evaluation of the effect of benznidazole compared with placebo or no treatment on the response to therapy in adults according to clinical primary endpoint by type of study (only in those with >60 months follow-up period). RCT: Randomized Clinical Trial; OBS: prospective observational study. References: [42], [18], [47], [21].
(TIF)

S4 Fig Evaluation of the effect of benznidazole compared with placebo or no treatment on the response to therapy in children according to secondary parasitological endpoint by type of study. RCT: Randomized Clinical Trial; OBS: prospective observational study. References: [38], [41].
(TIF)

S5 Fig Evaluation of the effect of benznidazole compared with placebo or no treatment on the response to therapy in adult patients according to secondary parasitological endpoint by type of study. RCT: Randomized Clinical Trial; OBS: prospective observational study. References: [40], [21], [50], [29], [30], [51], [43], [46], [47].
(TIF)

S6 Fig Evaluation of the effect of benznidazole compared with placebo or no treatment on the response to therapy in adult patients according to secondary parasitological endpoint by type of study (only in those with >60 months follow-up period). RCT: Randomized Clinical Trial; OBS: prospective observational study. References: [43], [47], [21].
(TIF)
