# Supplementary material for: Use of benznidazole to treat chronic Chagas disease: An updated systematic review with a meta-analysis
Source: PLoS Negl Trop Dis. 2022 May 16;16(5):e0010386. doi: 10.1371/journal.pntd.0010386 (PMC9135346; doi:10.1371/journal.pntd.0010386)
Supplement: S1 Text — (DOCX) [file pntd.0010386.s001.docx]

**S1.Text.Search strategy**

| **Data base** | **Platform** | **Search date** | **Results** |
| --- | --- | --- | --- |
| Medline | Ovid | February 19, 2021 | 276 |
| Embase | Elsevier | February 19, 2021 | 462 |
| Cochrane | Wiley | February 19, 2021 | 64 |
| Lilacs | Lilacs | February 19, 2021 | 69 |
| **TOTAL WITH DUPLICATES** |  |  | 871 |
| **TOTAL WITHOUT DUPLICATES** |  |  | **664** |

**Ovid MEDLINE(R) ALL <1946 to February 17, 2021>**

1 exp Chagas Disease/ (13475)

2 exp Trypanosomiasis/ (22643)

3 exp Trypanosoma cruzi/ (12083)

4 chaga$.tw. (15246)

5 trypanosom$.tw. (34818)

6 1 or 2 or 3 or 4 or 5 (44856)

7 exp Trypanocidal Agents/ (21901)

8 (trypanocidal adj2 agent?).tw. (132)

9 antitrypanosom$.tw. (973)

10 anti-trypanosom$.tw. (867)

11 exp Nitroimidazoles/ (18682)

12 Nitroimidazole?.tw. (2615)

13 Benz?nidazole.tw. (1339)

14 Benzonidazole.mp. (894)

15 Radanil.mp. (4)

16 Radanil.tw. (3)

17 7 or 8 or 9 or 10 or 11 or 12 or 13 or 14 or 15 or 16 (41795)

18 6 and 17 (5829)

19 Randomized Controlled Trials as Topic/ (140582)

20 Randomized Controlled Trial/ (523269)

21 Random Allocation/ (104677)

22 Double-Blind Method/ (162463)

23 Single-Blind Method/ (29748)

24 Clinical Trial/ (527488)

25 clinical trial, phase i.pt. (21275)

26 clinical trial, phase ii.pt. (34207)

27 clinical trial, phase iii.pt. (17924)

28 clinical trial, phase iv.pt. (2045)

29 controlled clinical trial.pt. (94072)

30 randomized controlled trial.pt. (523269)

31 multicenter study.pt. (288566)

32 clinical trial.pt. (527488)

33 exp Clinical Trials as Topic/ (352582)

34 (clinical adj trial$).tw. (392494)

35 ((singl$ or doubl$ or treb$ or tripl$) adj (blind$3 or mask$3)).tw. (178592)

36 Placebos/ (35334)

37 placebo$.tw. (223116)

38 randomly allocated.tw. (30501)

39 (allocated adj2 random$).tw. (33912)

40 19 or 20 or 21 or 22 or 23 or 24 or 25 or 26 or 27 or 28 or 29 or 30 or 31 or 32 or 33 or 34 or 35 or 36 or 37 or 38 or 39 (1698366)

41 Epidemiologic Studies/ (8564)

42 exp Case-Control Studies/ (1143646)

43 exp Cohort Studies/ (2091935)

44 Case control.tw. (131834)

45 (cohort adj (study or studies)).tw. (228770)

46 Cohort analy$.tw. (8823)

47 (Follow up adj (study or studies)).tw. (50737)

48 (observational adj (study or studies)).tw. (118324)

49 Longitudinal.tw. (261742)

50 Retrospective.tw. (576048)

51 Cross sectional.tw. (385326)

52 Cross-Sectional Studies/ (354025)

53 41 or 42 or 43 or 44 or 45 or 46 or 47 or 48 or 49 or 50 or 51 or 52 (3176687)

54 40 or 53 (4436298)

55 18 and 54 (567)

56 exp Animals/ (23826038)

57 exp Humans/ (19036693)

58 56 not 57 (4789345)

59 55 not 58 (440)

60 (202005* or 202006* or 202007* or 202008* or 202009* or 202010* or 202011* or 202012* or 202101* or 202102*).ed. (806310)

61 59 and 60 (13)

**EMBASE (ELSEVIER)**

#57 #17 AND #55 AND [19-5-2020]/sd NOT [20-2-2021]/sd 50

#56 #17 AND #55 719

#55 #37 OR #43 OR #54 4288435

#54 #46 OR #47 OR #48 OR #49 OR #50 OR #51 OR #52 OR #53 1732951

#53 ('cross sectional' NEAR/3 (study OR studies)):ab,ti 270140

#52 (epidemiologic* NEAR/3 (study OR studies)):ab,ti 122162

#51 (observational NEAR/3 (study OR studies)):ab,ti 213956

#50 ('follow up' NEAR/3 (study OR studies)):ab,ti 96880

#49 (case NEAR/2 control NEAR/3 (study OR studies)):ab,ti 134807

#48 (cohort NEAR/3 (study OR studies)):kw 15131

#47 'cohort analysis'/de 574867

#46 #44 NOT #45 592528

#45 'randomized controlled trial (topic)'/de 178604

#44 'prospective study'/de 598838

#43 #38 OR #39 OR #40 OR #41 OR #42 1353978

#42 'retrospective study'/de 912935

#41 'longitudinal study'/de 138866

#40 'family study'/de 25839

#39 'case control study'/de 153859

#38 'clinical study'/de 154269

#37 #18 OR #19 OR #20 OR #21 OR #22 OR #23 OR #24 OR #25 OR #26 OR #27 OR #28 OR #29 OR #30 OR #31 OR #32 OR #33 OR #34 OR #35 OR #36 2326444

#36 'prospective study'/de 598838

#35 placebo$:ab,ti 306167

#34 ((treble OR triple) NEAR/3 blind*):ab,ti 1299

#33 (double NEAR/3 blind*):ab,ti 210293

#32 (single NEAR/3 blind*):ab,ti 29333

#31 (random* NEAR/2 allocat*):ab,ti 42650

#30 rct:ab,ti 37081

#29 (randomi?ed NEAR/3 controlled NEAR/3 trial$):ab,ti 273566

#28 'placebo'/de 356119

#27 'crossover procedure'/de 62851

#26 'double blind procedure'/de 172147

#25 'single blind procedure'/de 38725

#24 'randomization'/exp 86560

#23 'phase 4 clinical trial'/de 3860

#22 'phase 3 clinical trial'/de 46534

#21 'multicenter study'/de 248391

#20 'controlled clinical trial'/de 429995

#19 'randomized controlled trial (topic)'/de 178604

#18 'clinical trial'/de 982122

#17 #6 AND #16 7936

#16 #7 OR #8 OR #9 OR #10 OR #11 OR #12 OR #13 OR #14 OR #15 212313

#15 radanil:ab,ti,kw 3

#14 benz$nidazole:ab,ti,kw 1538

#13 'benznidazole'/exp 2668

#12 nitroimidazole$:ab,ti 3149

#11 'nitroimidazole derivative'/exp 170069

#10 'anti trypanosom*':ab,ti 946

#9 antitrypanosom*:ab,ti 1079

#8 (trypanocidal NEAR/2 agent$):ab,ti 155

#7 'antitrypanosomal agent'/exp 41513

#6 #1 OR #2 OR #3 OR #4 OR #5 50754

#5 trypanosom*:ab,ti 36650

#4 chaga*:ab,ti 17271

#3 'trypanosoma cruzi'/exp 15580

#2 'trypanosomiasis'/exp 27185

#1 'chagas disease'/exp 16663

**COCHRANE LIBRARY (WILEY)**

#1 MeSH descriptor: [Chagas Disease] explode all trees 106

#2 MeSH descriptor: [Trypanosomiasis] explode all trees 132

#3 MeSH descriptor: [Trypanosoma cruzi] explode all trees 28

#4 (chaga?):ti,ab,kw 252

#5 (trypanosom*):ti,ab,kw 150

#6 {OR #1-#5} 323

#7 MeSH descriptor: [Trypanocidal Agents] explode all trees 46

#8 (trypanocidal near/2 agent?):ti,ab,kw 47

#9 (antitrypanosom*):ti,ab,kw 7

#10 (anti-trypanosom*):ti,ab,kw 4

#11 MeSH descriptor: [Nitroimidazoles] explode all trees 2740

#12 (Nitroimidazole?):ti,ab,kw 360

#13 (Benz?nidazole):ti,ab,kw 71

#14 (Radanil):ti,ab,kw 1

#15 {OR #7-#14} 2885

#16 #6 AND #15 with Cochrane Library publication date Between May 2020 and Feb 2021, in Trials 4

**LILACS. Portal Regional de la BVS (English)**

(tw:(chaga* OR trypanosom*)) OR (mh:("chagas disease")) AND (tw:(benzonidazole OR benznidazole OR trypanocidal agents OR antitrypanosom*)) OR (tw:("benzonidazole")) AND (tw:(randomi* OR rct OR trial*)) AND (year_cluster:[2020 TO 2021])
