# Supplementary material for: Use of benznidazole to treat chronic Chagas disease: An updated systematic review with a meta-analysis
Source: PLoS Negl Trop Dis. 2022 May 16;16(5):e0010386. doi: 10.1371/journal.pntd.0010386 (PMC9135346; doi:10.1371/journal.pntd.0010386)
Supplement: S2 Text — (DOCX) [file pntd.0010386.s002.docx]

**S2.Text. Newcastle-Ottawa quality assessment scale for cohort studies**

|  | Selection | | | | Comparability | | Outcome | | |
| --- | --- | --- | --- | --- | --- | --- | --- | --- | --- |
| Study | Representativeness of the exposed cohort | Selection of the non-exposed cohort | Ascertainment of exposure | Demonstration that outcome of interest was not present at start of study | Adjust for the most important risk factors | Adjust for other risk factors | Assessment of outcome | Follow up length | Loss to follow up rate |
| Catalioti 1998 [1] | - | * | * | * | - | - | - | * | - |
| Lauria-Pires 2000 [2] | * | * | - | * | * | - | - | * | * |
| Gallerano 2000 [3] | - | * | * | * | * | - | * | - | - |
| Streiger 2004 [4] | * | * | * | * | - | - | * | * | - |
| Viotti 2006 [5] | * | * | * | * | * | * | * | * | - |
| De Castro 2006 [6] | - | * | * | * | - | - | * | * | - |
| Fabbro 2007 [7] | * | * | * | - | - | - | * | * | * |
| Viotti 2011 [8] | * | * | * | * | * | - | * | * | - |
| Bertocchi 2013 [9] | - | * | * | * | - | - | * | - | - |

References:

1. Catalioti F, Acquatella H. Comparación de mortalidad durante seguimiento por 5 años en sujetos con enfermedad de Chagas crónica con y sin tratamiento de benznidazol. Rev Pat Trop. 1998; 29–31.

2. Lauria-Pires L, Teixeira ARL, Simões-Barbosa A, Vexenat AC, Tinoco DL, Nitz N, et al. Progressive chronic Chagas heart disease ten years after treatment with anti-Trypanosoma cruzi nitroderivatives. Am J Trop Med Hyg. 2000;63: 111–118. doi:10.4269/ajtmh.2000.63.111

3. Gallerano RR, Sosa RR. [Interventional study in the natural evolution of Chagas disease. Evaluation of specific antiparasitic treatment. Retrospective-prospective study of antiparasitic therapy]. Rev la Fac Ciencias Médicas (Córdoba, Argentina). 2000;57: 135–162. Available: http://eutils.ncbi.nlm.nih.gov/entrez/eutils/elink.fcgi?dbfrom=pubmed&id=12934232&retmode=ref&cmd=prlinks

4. Streiger ML, Barco ML del, Fabbro DL, Arias ED, Amicone NA. Estudo longitudinal e quimioterapia específica em crianças, com doença de Chagas crônica, residentes em área de baixa endemicidade da República Argentina. Rev Soc Bras Med Trop. 2004;37: 365–375. doi:10.1590/S0037-86822004000500001

5. Viotti R, Vigliano C, Lococo B, Bertocchi G, Petti M, Alvarez MG, et al. Long-Term Cardiac Outcomes of Treating Chronic Chagas Disease with Benznidazole versus No Treatment. Ann Intern Med. 2006;144: 724. doi:10.7326/0003-4819-144-10-200605160-00006

6. de Castro AM, Luquetti AO, Rassi A, Chiari E, da Cunha Galvão LM. Detection of parasitemia profiles by blood culture after treatment of human chronic Trypanosoma cruzi infection. Parasitol Res. 2006;99: 379–383. Available: http://link.springer.com/10.1007/s00436-006-0172-5

7. Fabbro DL, Streiger ML, Arias ED, Bizai ML, del Barco M, Amicone NA. Trypanocide treatment among adults with chronic Chagas disease living in Santa Fe city (Argentina), over a mean follow-up of 21 years: parasitological, serological and clinical evolution. Rev Soc Bras Med Trop. 2007;40: 1–10. Available: http://eutils.ncbi.nlm.nih.gov/entrez/eutils/elink.fcgi?dbfrom=pubmed&id=17486245&retmode=ref&cmd=prlinks

8. Viotti R, Vigliano C, Alvarez MG, Lococo B, Petti M, Bertocchi G, et al. Impact of Aetiological Treatment on Conventional and Multiplex Serology in Chronic Chagas Disease. Costa Santiago H da, editor. PLoS Negl Trop Dis. 2011;5: e1314. Available: http://dx.plos.org/10.1371/journal.pntd.0001314

9. Bertocchi GL, Vigliano CA, Lococo BG, Petti MA, Viottib RJ. Clinical characteristics and outcome of 107 adult patients with chronic Chagas disease and parasitological cure criteria. Trans R Soc Trop Med Hyg. 2013;107: 372–376. doi:10.1093/trstmh/trt029
