# Supplementary figures and images for: Use of benznidazole to treat chronic Chagas disease: An updated systematic review with a meta-analysis
Source: PLoS Negl Trop Dis. 2022 May 16;16(5):e0010386. doi: 10.1371/journal.pntd.0010386 (PMC9135346; doi:10.1371/journal.pntd.0010386)

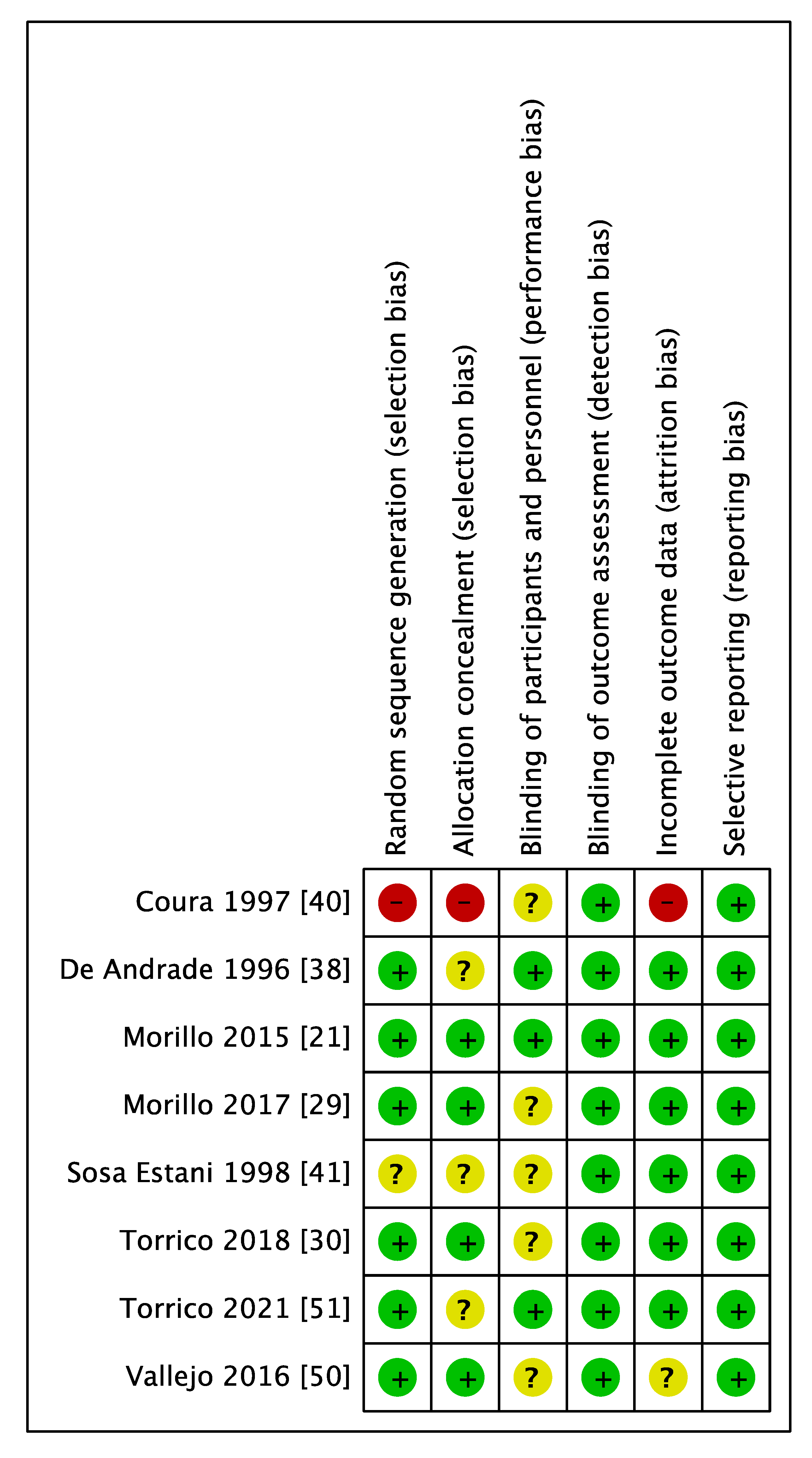

Supplement: S1 Fig — References: [40], [38], [21], [29], [41], [30], [51], [50]. (TIF) [file pntd.0010386.s003.tif]

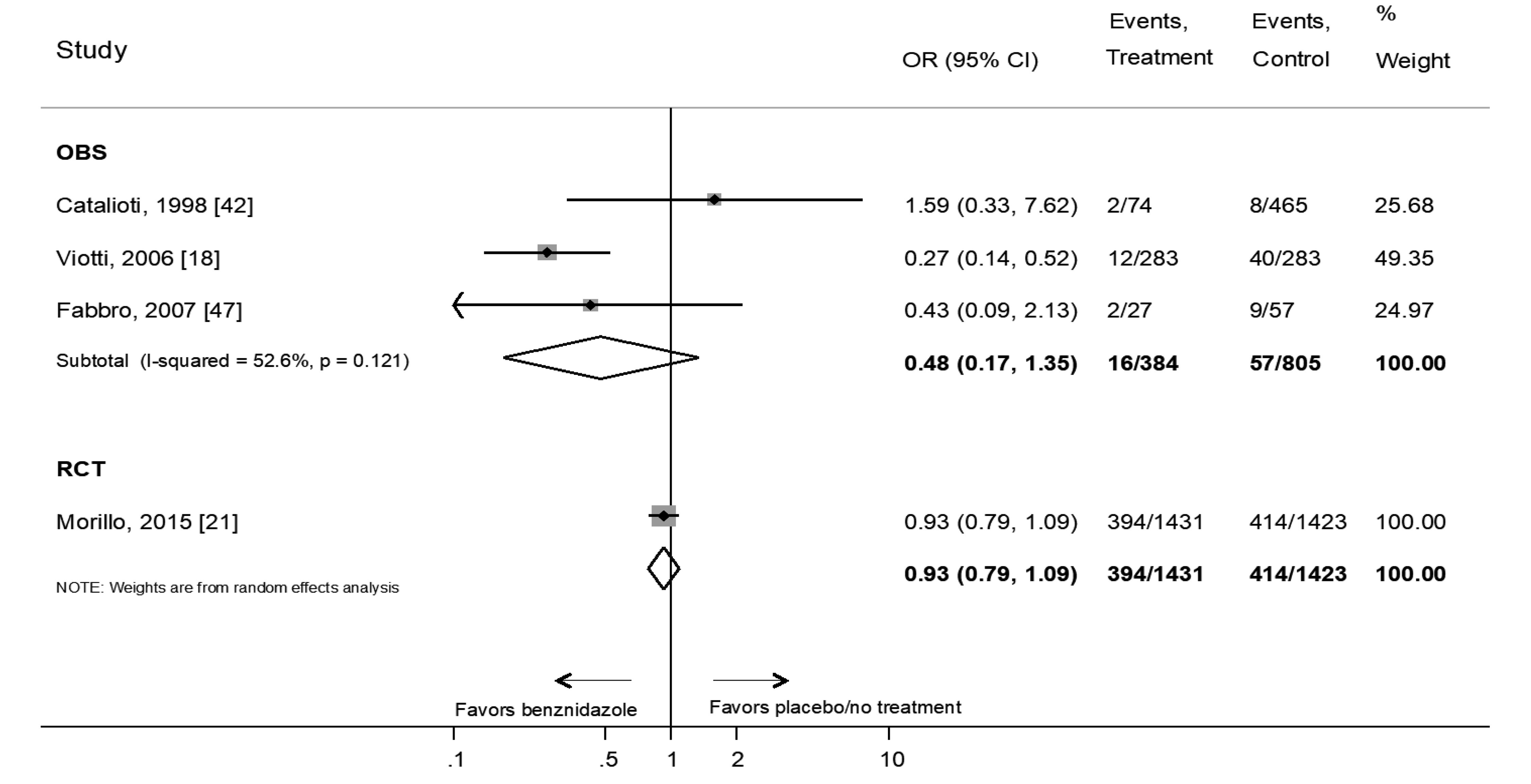

Supplement: S3 Fig — RCT: Randomized Clinical Trial; OBS: prospective observational study. References: [42], [18], [47], [21]. (TIF) [file pntd.0010386.s005.tif]

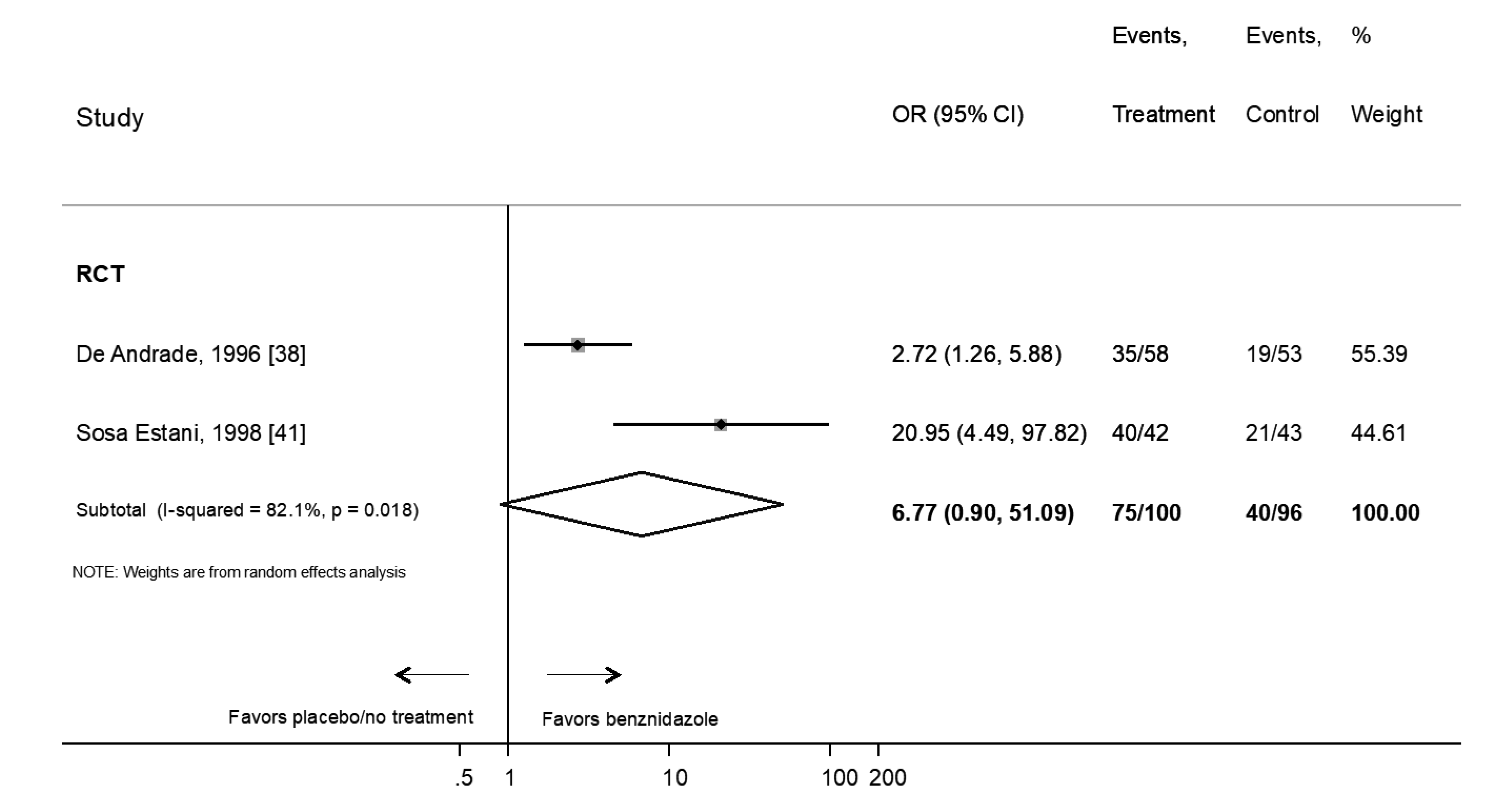

Supplement: S4 Fig — RCT: Randomized Clinical Trial; OBS: prospective observational study. References: [38], [41]. (TIF) [file pntd.0010386.s006.tif]

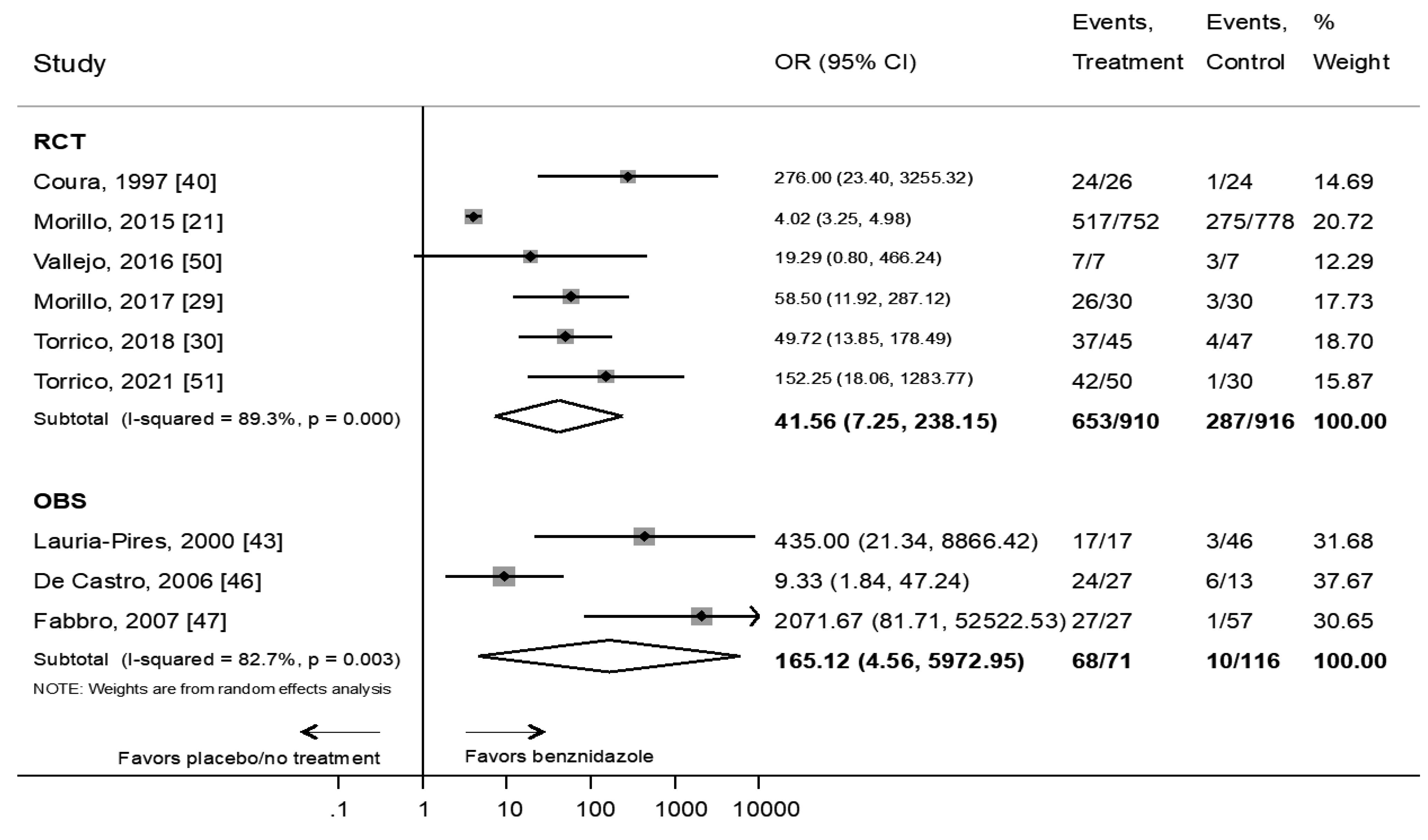

Supplement: S5 Fig — RCT: Randomized Clinical Trial; OBS: prospective observational study. References: [40], [21], [50], [29], [30], [51], [43], [46], [47]. (TIF) [file pntd.0010386.s007.tif]

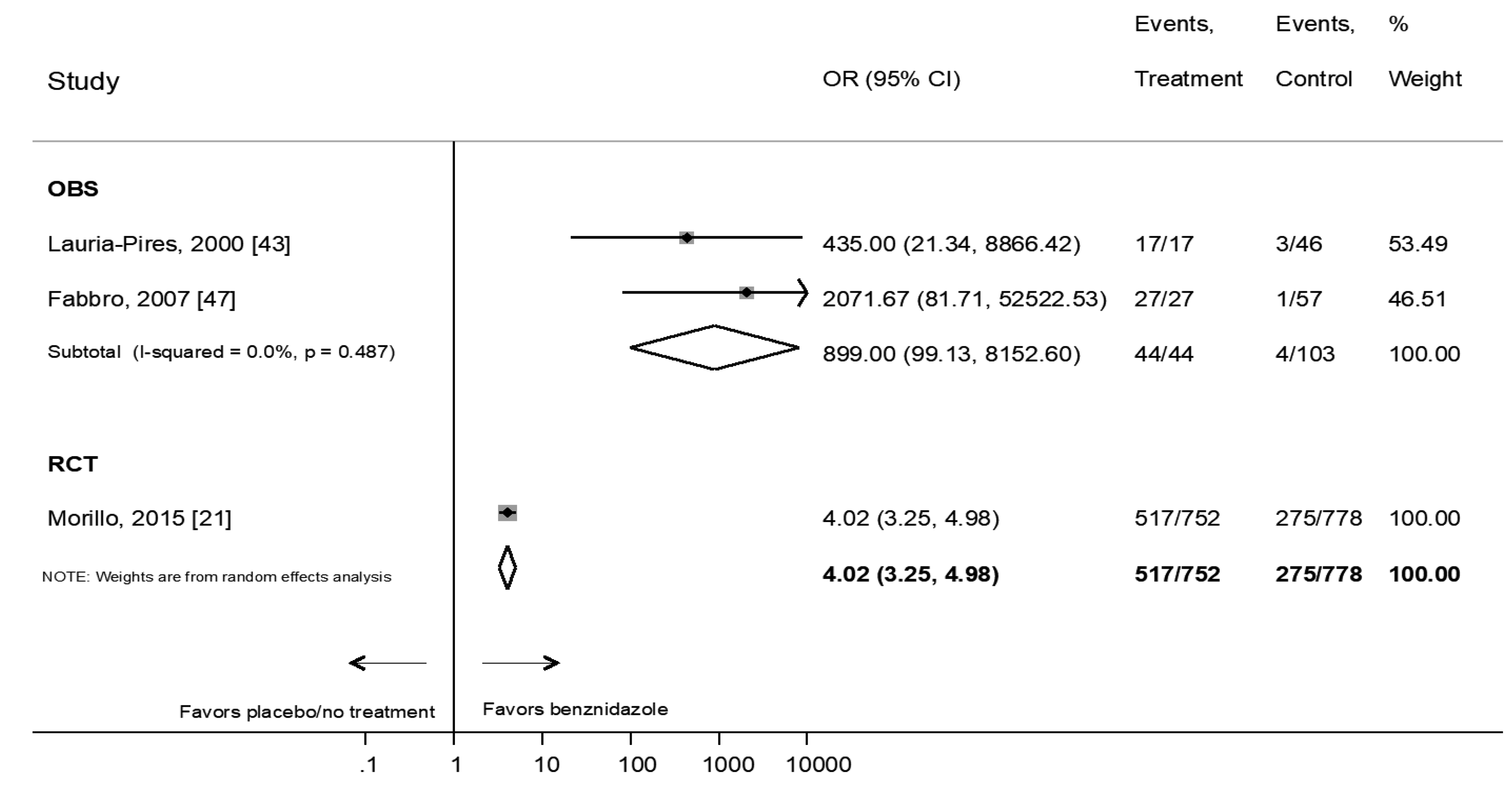

Supplement: S6 Fig — RCT: Randomized Clinical Trial; OBS: prospective observational study. References: [43], [47], [21]. (TIF) [file pntd.0010386.s008.tif]
